# Don’t stress: a case report of regional anesthesia as the primary anesthetic for gynecologic surgery in a patient with mitochondrial myopathy and possible malignant hyperthermia susceptibility

**DOI:** 10.1186/s12871-019-0909-1

**Published:** 2019-12-14

**Authors:** Marci B. Pepper, Catherine Njathi-Ori, Michelle Ochs Kinney

**Affiliations:** Mayo Clinic Department of Anesthesiology and Perioperative Medicine, 200 First Street SW, Rochester, MN 55905 USA

**Keywords:** Regional anesthesia, Combined spinal epidural, Mitochondrial myopathy, Malignant hyperthermia

## Abstract

**Background:**

We aim to describe the evaluation and management of a patient with the uncommon combination of both mitochondrial myopathy and possible malignant hyperthermia susceptibility as an important source of information and as a valuable example of the role of regional anesthesia for patients with these diagnoses.

**Case presentation:**

A 24 year old woman with a history of possible mitochondrial myopathy and possible malignant hyperthermia susceptibility presented for gynecologic surgery. Surgery was well tolerated with combined spinal epidural anesthesia as well as sedation with midazolam, ketamine, and fentanyl.

**Conclusions:**

Anesthetic management of patients with mitochondrial myopathy is challenging, made even more so with concurrent malignant hyperthermia susceptibility. This case adds an example to the literature of employing regional anesthesia as a safe approach to this complex care.

## Background

Anesthetic management of patients with mitochondrial myopathy (MM) is challenging: the incidence is low, the phenotype and comorbidities are heterogeneous, every organ system may be involved, and evidence based guidelines are lacking [1]. Recent review articles provide helpful discussion of principles of perioperative management of patients with MM [1–3]. A thorough preoperative evaluation to identify and qualify severity of systemic involvement is required. Care must be taken to avoid tipping the balance of energy supply and demand, including checking baseline pH and lactate levels followed by perioperative monitoring [1, 3]. Since these patients may have defects in energy production and use, they should have minimization of fasting times and perioperative monitored glucose supplementation to avoid both hypo- and hyperglycemia [1–3]. Metabolic demands should be minimized, including avoidance of stress, pain, nausea and vomiting, and hypoxemia, and maintenance of normothermia [1–3]. Preparations ought to be made for possible increased sensitivity to anesthetic and analgesic agents and neuromuscular blockade [1–3]. Although the safety of propofol use in patients with MM is unsettled, and a single bolus dose is likely tolerated, avoidance of propofol-based total intravenous anesthetic (TIVA) is recommended as it may lead to propofol infusion syndrome [1–3]. Patients with MM are unlikely to truly have malignant hyperthermia (MH); however, succinylcholine administration in myopathic patients may cause hyperkalemia or anesthesia induced rhabdomyolysis, so it should be avoided [1]. Regional anesthesia should be used whenever possible.

While these principles are helpful and these review articles belong in the anesthesiologist’s armamentarium, in the setting of minimal evidence-based guidelines, case reports continue to be valuable in guiding and providing examples of management of these complex patients with sometimes unclear diagnoses. Thus, we present a case of a patient with both MM and MH susceptibility managed with regional anesthesia and sedation for a gynecologic surgery. Written authorization from the patient was provided for submission of a case report.

## Case presentation

A 24 year-old, 95 kilogram (Kg), 165 centimeter (cm) woman was scheduled for a diagnostic laparoscopy for an indeterminate pelvic mass. Historical workup more than 5 years prior for congenital hypotonia, delayed acquisition of motor skills, persistent discoordination, and chronic fatigue included a muscle biopsy with pathologic examination and oxidative phosphorylation enzymology as well as a mitochondrial deoxyribonucleic acid (DNA) and multi-gene panel for cellular energetic defects including 656 genes. Skeletal muscle oxidative phosphorylation enzymology was equivocal for a possible complex 1 defect and decreased enzymatic activity of complex 1 was observed. Although no associated mitochondrial DNA defect was identified, she acquired a diagnosis of possible MM. Gene sequencing revealed a variant of uncertain significance, reported with conflicting predictions of “possibly damaging” and “tolerated,” in the ryanodine receptor 1 (RYR1) gene (Position Chromosome 19: 38955314, Exon 23, Variant c.[2822C > T]), suggestive of possible MH susceptibility. There was no family history of MH. Additional studies included normal electromyography, brain magnetic resonance imaging, electrocardiogram, and transthoracic echo. Her past medical history was otherwise notable for fibromyalgia/chronic musculoskeletal pain, possible spondyloarthropathy, endometriosis, adnexal cyst, and ectopic pregnancy. She had undergone prior surgical procedures with neuraxial or peripheral nerve blockade. She was warned by medical providers elsewhere to avoid propofol, lactated Ringer’s solution, and succinylcholine.

Given this history, we favored regional anesthesia and the surgical service agreed to an open rather than the originally scheduled laparoscopic procedure. She was pre-medicated with 1 gram (g) oral acetaminophen and intravenous (IV) fentanyl 50 micrograms (mcg) and midazolam 3 milligrams (mg). A combined spinal epidural procedure was performed with the patient sitting upright and with midline approach at lumbar interspace 3–4 with an 18 gauge Tuohy needle, 27 gauge Whitacre needle, and 20 gauge epidural catheter. Loss of resistance with preservative-free normal saline was obtained at 6 cm. Intrathecal injection of 2 milliliters (mL) 0.75% bupivacaine with 8.25% dextrose, 15 mcg fentanyl, and 200 mcg epinephrine was performed. The epidural catheter threaded easily and a test dose of 3 mL of 1.5% lidocaine with 5 mcg/mL epinephrine was negative for intrathecal or intravascular catheter location. Approximately 30 min later, the patient had a dermatomal level around thoracic (T) spinal nerve T8, so the catheter was bolused with 10 mL of 2% lidocaine. After confirming T6 dermatomal level, incision was made. Anesthesia was maintained with divided doses of 10 mL 0.5% bupivacaine with 5 mcg/mL epinephrine and 10 mL 2% lidocaine. For sedation, in addition to previously mentioned pre-medications, she received IV divided doses of 30 mg ketamine, 9 mg midazolam, and 550 mcg fentanyl. For antiemetics she received 0.625 mg droperidol IV, 4 mg dexamethasone IV, and 4 mg ondansetron IV. She was maintained on a 5% dextrose and normal saline IV infusion at 100 mL/hour with intraoperative point-of-care glucose monitoring. Baseline and perioperative lactates were not monitored, although in retrospect we would include this laboratory evaluation. Skin temperature was monitored and normothermia was maintained with a forced air warmer. Surgical time was 4 h and included Pfannenstiel incision, lysis of adhesions, and left ovarian cystectomy. At the conclusion of the case the surgeons infiltrated the wound with a solution of 20 mL 1.3% liposomal bupivacaine in 100 mL normal saline and the epidural catheter was removed. The procedure was well tolerated without complications. The patient was discharged home on postoperative day three.

## Discussion & Conclusions

When a patient presents for surgery with a history suggestive of MM, the diagnostic features of this spectrum of disorders make anesthetic evaluation, planning, and decisions difficult. Although there are some well characterized mitochondrial myopathic syndromes, some genetic mutations have incredibly variable clinical presentations and some similar clinical presentations can be caused by different genetic mutations [1]. Moreover, although massive parallel or next-generation genetic sequencing methodologies have emerged as a new gold-standard for accurate diagnosis of mitochondrial DNA disorders [3], their increased availability and use can reveal genetic mutations of uncertain clinical significance, particularly to the anesthesiologist making assessments in a brief perioperative window. History of muscle biopsy with pathology and biochemical testing can add diagnostic clarity, but these tests might have limited sensitivity and specificity and add insufficient information to aid in formulation of an anesthetic plan [3]. The increased availability of gene sequencing has implications for evaluating patients with possible MH susceptibility, as well. Patients may present with a list of results, frequently including variants of uncertain significance, without corresponding interpretation or recommendations from medical geneticists or genetic counselors. Our approach is to apply MH precautions and non-triggering anesthetics to these patients unless their results have been clearly interpreted by a qualified provider as benign. Our patient had laboratory variations suggestive of possible MM and an indeterminate RYR1 defect suggestive of possible MH susceptibility. We aimed to presumptively optimize her care.

Even in the setting of diagnostic clarity, anesthetic management of patients with MM can be challenging because of the wide and variable degree of systemic involvement. Elucidating quality and severity of individual patient signs and symptoms preoperatively, which can include cardiomyopathy, arrhythmias, respiratory or skeletal muscle weakness, dysphagia, risk for rhabdomyolysis, seizures, neuropathy, and kidney and liver dysfunction, among other relevant considerations for the anesthesiologist, is paramount [1]. Our patient had primarily musculoskeletal complaints without any objective evidence of widespread systemic derangements, posing fewer limitations on our anesthetic plan than might be the case with other patients with MM.

Although patients with MM ought not to get succinylcholine due to risk of hyperkalemia or anesthesia induced rhabdomyolysis, they are not considered to be at higher risk than average for developing true MH. However, the two diseases can coexist, and this very uncommon coexistence was a concern in our patient with an indeterminate RYR1 mutation. These concurrent laboratory variations added a layer of complexity to her care and pushed us strongly in favor of a primarily regional anesthetic. In addition to following the aforementioned perioperative recommendations regarding the balancing of metabolic supply and demand and precautions about sensitivity to anesthetic and analgesic agents, we chose to avoid MH triggering agents. Although controversial, avoidance of propofol in MM is recommended because of increased risk of propofol infusion syndrome [1]. Essentially all anesthetic agents depress mitochondrial function and many are potent inhibitors of complex 1 [2]; however, there are reports describing successful general anesthetics for patients with both MM and MH with induction with ketamine, methohexital, and fentanyl followed by maintenance with methohexital, dexmedetomidine, and sufentanil infusions [4], induction with midazolam, fentanyl, and ketamine followed by maintenance with ketamine infusion and inhaled nitrous oxide [5], and induction with fentanyl and etomidate followed by maintenance with remifentanil and dexmedetomidine infusions and inhaled nitrous oxide [5]. In summary, there is no consensus regarding an ideal anesthetic for these patients, in part because patients with these disorders are highly variable, but a number of parenteral agents and nitrous oxide have been used judiciously and safely [2]. Nevertheless, regional anesthesia is preferable when possible, so we proceeded with neuraxial technique. We considered dexmedetomidine sedation, but our patient desired maximal amnesia and hypnosis and had no evidence of respiratory dysfunction. As a backup plan, preparations were made for conversion to a non-MH triggering general anesthesia with induction with fentanyl and ketamine, neuromuscular blockade with rocuronium, maintenance with dexmedetomidine and ketamine infusions with bolus dosing of midazolam and fentanyl, and monitoring of anesthetic depth with a BIS monitor. We planned to avoid nitrous oxide to avoid distension of the abdominal viscera. Thus, we present a case of surgical anesthesia for an open pelvic operation with combined spinal epidural as well as sedation with midazolam, ketamine, and fentanyl that was well tolerated in a patient with possible MM and MH. Our case adds an example to the literature of a safe approach to evaluation and management of a complex patient with an uncommon combination of comorbidities with competing management priorities.

## Data Availability

Not applicable.

## Abbreviations

cm: Centimeter
DNA: Deoxyribonucleic acid
g: Gram
IV: Intravenous
Kg: Kilogram
mcg: Micrograms
mg: Milligrams
MH: Malignant hyperthermia
mL: Milliliters
MM: Mitochondrial myopathy
RYR1: Ryanodine receptor 1
T: Thoracic spinal nerve
TIVA: Total intravenous anesthetic
